# Bridge-Work

**Published:** 1898-01

**Authors:** J. L. Young


### THE DENTAL REGISTER.

Vol. LII.] JANUARY, 1898. [No. 1.

### COMMUNICATIONS.

### Bridge-Work.

J. L. YOUNG, D.D.S.

Read before the Detroit Dental Society on November 13, 1897.

*Mr. President and Gentlemen of the Detroit Dental Society:*

In consenting, at the first regular meeting, to read a paper before this society in November, I was prompted to make the effort by the example set by our worthy President and Secretary, and also by the fact that I consider it an honor to be asked to read a paper before such a learned body. Every member of this society should consider it an honor, as well as his duty, to do all in his power to advance the interests of the society.

In selecting a subject to write on with the hope of interesting and possibly calling forth some discussion, I could think of nothing better than Bridge-Work. It will be of no value to take up your time with the history and development of this special branch of our profession, but to proceed at once to point out what seems to the writer to be some important points which are often overlooked in this work.

Before going to this, however, it might be well to compare bridges with partial plates, as it is in such cases that bridge-work is practical if the patient can afford it. If we had all attained that high standard, that, regardless of fees, all operations should be so performed as to be of most service to the patient, then there would be few plates inserted in cases suitable for bridge-work.

When we consider the impossibility of getting an accurate impression of many cases requiring partial dentures and the illfitting appliances made, causing undue pressure on the soft parts around the remaining teeth with the results of receded gums and decay above the enamel margin, and then, too, the unnatural feeling of such an appliance, with the continual annoyance of particles of food working between them and the sensitive soft tissues, is it any wonder we say, "Well done," when a properly-constructed bridge is cemented firmly in place, supplying the lost organs almost as firmly and comfortably as the natural ones were before attacked by decay?

The following advantages are also claimed for this work over partial plates:

First—The absence of any mechanical appliance to interfere with the tongue in articulation, mastication, deglutition and the sense of taste.

Second—The remaining teeth are not abraded by clasps.

Third—The soft parts are not covered up and inflammation induced.

And, Fourth—The solidity and immovability of the supplied organs, at all times.

The architect, in constructing a bridge, first considers the possible strength of the abutments and piers, so, too, in dental bridge-work these points must be first considered to see if the strain on the finished piece will be properly borne by the supports.

It might be well to consider here the possible strength of the muscles of mastication. According to Black, it ranges from thirty to one hundred and seventy-five pounds in the incisor region and from sixty to two hundred and seventy in the molar region. While this may seem excessive force, it is the opinion of the writer that it is far below the possible under favorable conditions. An excessive bite of this kind on the natural organs is very likely to come on one or two teeth of each jaw, whereas on a bridge of the entire upper denture supported by the second molars and cuspids the strain would be divided among four very strong teeth held firmly together. This is claimed to be one very strong argument in favor of bridge-work.

Properly speaking, a bridge must have two abutments, but this does not always apply in dental bridge-work. For example, a missing lateral can be successfully supported by the cuspid or a second bicuspid by a first molar. Most all other cases must have two or more attachments.

As we have a great many systems of bridge-work, it is the opinion of the writer that it is best to select one of these and become so accustomed to its use as to be able to apply it to any case. The Morrison System, with some modifications, has been used by the writer for some years with good results.

The attachments to the abutment teeth should be so constructed as to do away with any possibility of solution of the cement and subsequent loosening of the piece. Therefore bands or open-faced caps can not be employed with the hope of having a permanent piece of work, and much less dove-tailed bars anchored in fillings. Besides, the majority of teeth are so shaped as to render it impossible to properly fit a band at the gingival margin without so grinding the tooth as to utterly spoil its appearance and also leave portions of the tooth from which the enamel has been removed exposed to the fluids of the mouth. We are thus confined to two forms of attachments, the full caps and the Richmond crowns, the latter to be used where too much show of gold would be unsightly.

To properly cap a tooth it is essential that it be so shaped that the circumference at the gingival margin be at least as great as at any point between it and the occlusal portion of the tooth. Where it is possible, it is even better to so shape the tooth that the circumference at the gingival margin is considerably in excess of any other point.

To show how to do this on paper is very simple, but to perform the operation in many cases is very difficult and often is the cause of failure in an otherwise good piece of work. It might not be amiss to give here the method of the writer in constructing attachments for a bridge running from the cuspid to the second molar superior.

If the outlines of the molar are complete, first take an impression in plaster, if necessary, in two sections, then make a cast of plaster. When this is thoroughly set remove the cast and cut the tooth off a little above the gum margin. With a true carborundum in the engine cut down the cusps of the tooth so as to allow at least one thirty-second of an inch at the nearest point between the abutment tooth and the occluding teeth. With a diamond disk, kept wet, cut the approximal surfaces of the tooth slightly slanting toward the occlusal surface. With a small, true carborundum slant the buccal and lingual walls as the approximal ones. Now comes the hard part, especially if the third molar is in position, to properly shape the disto-buccal and disto-lingual corners, and also to remove the bulge of enamel beneath the gum margin on the lingual and buccal surfaces. A diamond disk, swedged saucer shape, is of great service in these cases, as it is a rapid cutter when kept wet and does not wear thick and injure the gums.

The Case Enamel Cleavers and Evan's Taper Bur Root Trimmers are also a great help. With binding wire in a dentimeter twist till tight at the gingival portion, and if the tooth is properly trimmed down the wire can be easily removed, showing the shape of the tooth and also giving the circumference. The plaster tooth is now trimmed at the gingival portion so that the loop of wire will just fit over it. With the box and screw appliance of the Morrison System, get an impression of the plaster tooth in two parts of Molott's metal. In this can be swedged a perfect seamless cap, exactly reproducing the tooth in gold. The cusps are now filled in with 22 K. solder (if 22 K. gold has been used), the top of cap trimmed to conform with the gum line and then beveled. The cap is then forced on the tooth giving a perfect fit and articulation and no irritation to the gums.

A full cuspid cap can be made in the same way.

If the cuspid possesses a vital pulp and it is decided to use a Richmond crown; with a diamond disk, nick the tooth well up to the gum on both lingual and labial sides, and then adjust rubber dam and with excising forceps remove the crown exposing the pulp. Having previously prepared the hypodermic syringe with a solution of cocaine, immediately insert the needle into the exposed pulp, and at the same time force some of the solution into it. In this way the needle can be forced well up and the pulp thoroughly numbed with very little pain, as the pulp has not yet recovered from the shock of excising the tooth. With a Donaldson cleanser now remove the pulp, and, if hemorrhage can be controlled, the root may be filled at once by first using camphophenique, and then chlora-percha and gutta-percha cones, entirely filling the canal. In reaming out the canal for the post, the gutta-percha acts as a guide and lessens the danger of perforating the root.

At the following sitting the root can be trimmed down by the use of the saucer-shaped diamond disk and the different root trimmers so as to fit the band, and the labial portion beveled off to a level with the gum. The measure of the root can be taken as with the molar, and then cut open so as to give the length to cut the piece out of pure gold No. 28 B. & S. gage. The width of this depends on the root, but one-eighth inch is generally sufficient. The ends should be beveled and lapped and united by 22 K. solder. The band should then be fitted to the root and ground to come flush with the end of it. To this is soldered the cap of pure platinum No. 30 B. & S. gage. The cap is now placed on the root and a hole punched into the pulp canal to receive the post. This should be of iridio-platinum No. 13 B. & S. gage. The root is now reamed out, the cap re-adjusted, and the post forced through the hole in the cap, being careful not to get the hole too large. Mark the post just below the cap, remove post, then cap, and place together and solder. Try it on the root to see that everything is right, then bevel the edge of the platinum cap to allow the porcelain facing to overlap, and thus prevent the show of metal. Cut the post off to admit of proper occlusion, take bite in wax and impression in plaster of the cuspid attachment and adjoining incisors. Run casts and mount on articulator in the usual way, select plate tooth and grind to fit, having it at least one thirty-second inch shorter than the finished crown is to be. To the tooth fit a backing of pure gold No. 32 B. & S. gage, allowing it to cover the entire back of the tooth, over this fit a platinum backing No. 30 B. & S. gage, to extend from start of bevel to point of tooth, and nick the pins to hold it on. Take small pieces of pure gold long enough to extend across the occlusal edge of tooth, and adapt to fit by pressing unvulcanized rubber and pressing the tooth into it. This is made fast to the backing with sticky wax, the gold coming flush with the labial surface of the tooth. To this is added more pieces of pure gold in the same manner till it is at least one thirty-second of an inch thick.

To prevent the porcelain being checked by the borax, it is coated with a varnish composed of four parts yellow ochre and one part boracic acid. The tooth is now inverted in plaster and sifted coal ashes. When set the wax is boiled out and the gold tip and backing united with 22 K. solder. When cool, the labial surface is trimmed up, the tooth adjusted on the cap and waxed in place. The facing is again varnished and invested. When set the wax is removed as before, and the cap and tooth united with 20 K. solder by heating the whole investment to such a degree that the solder flows without the use of a small flame. In this way the solder flows so as to entirely unite the cap and the backing of tooth. When cool the crown is trimmed to properly articulate and placed on root. A crown is thus obtained having the porcelain entirely protected with gold and with very little danger of cracking in soldering.

The cap and crown in position, a bite is taken in wax, then a plaster impression extending from the second molar to the lateral which should remove cap and crown with it—also a plaster impression of the opposing teeth. Casts are run and then mounted on articulator, and the dummy teeth supplied in the usual way. The bridge should be carefully finished so that when cemented on there are no pockets for lodgment of food.

The writer has frequently been taken to task for cutting down perfectly-sound teeth in order to fit attachments for a bridge, particularly where only one tooth is lost, as first lower molar. Is it not better to supply the lost organ, and in doing so forever protect the adjoining teeth from decay, than to allow the teeth to separate and interfere with proper occlusion, probably causing exostosis? Or to insert a plate to cause irritation of the gums and abrade the adjoining teeth, saying nothing of the annoyance and discomfort? If bridge-work is good at all it is in just such cases.

In the opinion of the writer a tooth properly capped, under ordinary conditions, is not liable to be troubled with decay if the pulp be alive, and if the pulp be dead there is no better way of preserving the tooth.

The extent to which bridge-work may be employed depends very largely on the condition of the supports. Where teeth are firmly set, and the surrounding tissues healthy the cuspids and second molars will successfully support a bridge of fourteen teeth. Where a bridge has but two attachments, it is well to have the intervening teeth as near the straight line as possible, and thus do away with the lateral strain. The third molar can often be used to good advantage as an abutment, and if firmly set will successfully carry a span to the cuspid of the same side.

DISCUSSION OF PAPER BY H. C. RAYMOND, D.D.S.

*Mr. President:*—Dr. Young's paper is what a paper on such a subject should be. It is practical and right to the point. It shows that the author knows well his subject and has written largely from experience. It also shows that he has decided opinions and is not afraid to express them.

The paper is an excellent one and well worthy of discussion. In championing bridge-work, the writer, I think, steps wide of the mark in his comparison between it and plate-work. Some of the advantages he claims for this work over plates are, that deglutition and the sense of taste are not interfered with, the teeth are not abraded by clasps, and inflammation is not caused in the soft parts by covering them. It is true that bridge-work has in some cases decided advantages over plate-work, but those named are not among them. As to charging such evils to the use of plates, I might with just as good reason condemn all bridge-work, because of the foul stench produced in the mouth, periostitis of the banded teeth, abrasion and decay beneath the bands, and numerous other evils, brought about by imperfect work. I would soon be met with the argument that bridge-work properly and scientifically constructed does not cause any of these evils, and I answer with equal reason that neither does plate-work when properly done. The soft parts are certainly covered with a plate, but when a *gold* plate fits perfectly and strict cleanliness is observed on the part of the wearer there is no inflammation of the parts covered. Clasps are seldom, if ever, necessary, and when they are, if properly adjusted, little or no abrasion is caused. Deglutition is not in any way interfered with, when correct principles are followed, and regarding the sense of taste being spoiled or interfered with it is only necessary for us to remember enough of our physiology to know that sense of taste lies principally in the tongue, its mucous membrane having the papillæ or taste buds located there, while the posterior portion of the palate is slightly supplied also with them; but that portion of the palate ought not to be covered by the plate. The sense of taste is partly illusory any way and is largely assisted by the sense of smell.

I object strongly to the way in which plate-work is condemned by some enthusiastic bridge-workers. I have removed many bridges from the mouth, and I have come across more foul mouths, abscessed, abraded and sensitive teeth, and inflamed soft parts from their use than all the plates in the city combined would cause. It almost inclines me to the opinion that the fewer bridges, especially large ones, that a dentist inserts, unless he be an expert, the better he will hold his patients, and the more will they remain his friends in the years to follow. But, with all this, bridge-work is one of the most important of the advances made in the profession and will certainly always be with us. In the hands of the few it will be a success, but those who are not expert mechanics ought not to attempt it. The writer of the evening has laid down some strong fundamental principles on which this work should be carried out, and his thoroughness is to be commended. His condemnation of bands and open-faced crowns can not be too strongly endorsed, for usually they are an abomination, and there are few cases in which their use is excusable. I must take issue, however, with Dr. Young on his views regarding anchoring bridges in fillings. There are places where such work is ideal; this applies usually to the insertion of one tooth. It is, of course, more often indicated where there are already cavities in the teeth adjoining the space. In some instances, however, I would not hesitate to make a cavity. I

have put several bridges in and call to mind now, one, a first superior molar, anchored in amalgam fillings, and a superior lateral anchored in gold fillings. The latter I saw only a short time since and it seemed perfect, though it has been in over six years; the other has been in nearly seven years, and is in equally good condition.

While I think of it, I wish to say a few words about capping teeth with living pulps in them. There is considerable controversy going on most of the time regarding it, and opinion is divided, though I believe some of the most experienced and thorough workers are in favor of removal of the pulps of all teeth capped. While I would not go quite so far as that, there are certainly strong reasons for doing so. When grinding down a pulpless tooth there is no excuse for not properly shaping it, but those who have done this work know the difficulties of preparing for capping a tooth with a living pulp in it. In many cases there is extreme sensitiveness after cutting through the enamel, and with a nervous patient one is only too apt to say "it will do," when he knows the work is not completed. One of the gravest faults of this work is imperfect preparation of the abutments. Then even if we succeed in shaping the tooth properly, the irritation caused by the grinding sometimes does not subside, and the result is that often after the work is completed congestion and death of the pulp follow. Of course the pulp chamber and canals can be opened through the cap, but it is not nearly so easy to fill the roots through such a small opening as must necessarily be made as it would be were the work done before capping. Then the patient is apt to think it was not quite right in the first place, and we devoutly wish we had destroyed and removed that pulp to begin with. I very much prefer to destroy the pulp myself, and remove it, rather than let it die and slough away to be removed piecemeal in a foul condition. Everything considered, I believe the painstaking, thoughtful operator will incline to my opinion that it is better in a large majority of cases to remove the pulp and fill the canals before using a tooth for attachment.

Dr. Young says he uses pure gold for his bands and backing of Richmond crowns. Pure gold is not strong enough for such work. Bridge-work requires strength above all things, as indicated by the immense pressure exerted in different directions during mastication. At least 22 K. gold ought to be used in its construction, and for some cases 20 K. gold is quite soft enough.

The care exercised in protecting the occluding edge of the porcelain teeth is very necessary, but I do not quite understand Dr. Young's reason for backing his teeth with pure gold, then platinum, etc.; it seems to me rather complicated, and I think equally as good results can be obtained by simpler and quicker methods. Regarding different methods of bridge-work it is perhaps best, as the writer of the paper says, to select some system and try and perfect one's self in it, or at any rate to follow out some definite line of procedure. Some systems that have recently come out, such as the Lowry and Mason, will probably simplify the work. But for success in this branch of dentistry one does not want to tie himself down to any one method; he must use, apply, or invent whatever is best for the particular case in hand. He must be a thinker, a man of keen judgment and discernment, and a *good* mechanic, and with all this he must be an artist. To insure success certain principles and practices must be followed. The piers must be parallel; in proportion as they are not, so will failure follow. Accuracy of occlusion is absolutely necesary. A healthy condition of roots or teeth used as abutments and of the surrounding parts is indispensable, and the fitting of the attachments must be perfect. Solder of as high a grade as possible should be used, and gold as pure as requirements for strength will permit. Receptacles where food can lodge must be carefully avoided, and the piece must be made as nearly self-cleansing as possible.

I am a strong advocate of bridge-work in its place. It is the thing in the hands of the right men, which means above all things careful discrimination in its use. Its coming in has indeed done much for us, and as Dr. Register writes in last month's *International Journal:* "It has undoubtedly raised the standard of dental mechanics, for it has brought us nearer perfection in mechanical technique than ever before."

It certainly has helped to raise the profession above the slipshod methods and cheap, unskilled work that has been in vogue since the rubber period came in, for a man whose limit is rubber plates had better not attempt bridge-work.

